# Reductions in Cortico-Striatal Hyperconnectivity Accompany Successful Treatment of Obsessive-Compulsive Disorder with Dorsomedial Prefrontal rTMS

**DOI:** 10.1038/npp.2015.292

**Published:** 2015-10-28

**Authors:** Katharine Dunlop, Blake Woodside, Marion Olmsted, Patricia Colton, Peter Giacobbe, Jonathan Downar

**Affiliations:** 1 Institute of Medical Sciences, University of Toronto, Toronto, ON, Canada; 2 MRI-Guided rTMS Clinic, University Health Network, Toronto, ON, Canada; 3 Department of Psychiatry, University Health Network, Toronto, ON, Canada; 4 Department of Psychiatry, University of Toronto, Toronto, ON, Canada; 5 Eating Disorders Program, University Health Network, Toronto, ON, Canada; 6 Toronto Western Research Institute, University Health Network, Toronto, ON, Canada

## Abstract

Obsessive-compulsive disorder (OCD) is a disabling illness with high rates of nonresponse to conventional treatments. OCD pathophysiology is believed to involve abnormalities in cortico-striatal-thalamic-cortical circuits through regions such as dorsomedial prefrontal cortex (dmPFC) and ventral striatum. These regions may constitute therapeutic targets for neuromodulation treatments, such as repetitive transcranial magnetic stimulation (rTMS). However, the neurobiological predictors and correlates of successful rTMS treatment for OCD are unclear. Here, we used resting-state functional magnetic resonance imaging (fMRI) to identify neural predictors and correlates of response to 20–30 sessions of bilateral 10 Hz dmPFC-rTMS in 20 treatment-resistant OCD patients, with 40 healthy controls as baseline comparators. A region of interest in the dmPFC was used to generate whole-brain functional connectivity maps pre-treatment and post treatment. Ten of 20 patients met the response criteria (⩾50% improvement on Yale-Brown Obsessive-Compulsive Scale, YBOCS); response to dmPFC-rTMS was sharply bimodal. dmPFC-rTMS responders had higher dmPFC-ventral striatal connectivity at baseline. The degree of reduction in this connectivity, from pre- to post-treatment, correlated to the degree of YBOCS symptomatic improvement. Baseline clinical and psychometric data did not predict treatment response. In summary, reductions in fronto-striatal hyperconnectivity were associated with treatment response to dmPFC-rTMS in OCD. This finding is consistent with previous fMRI studies of deep brain stimulation in OCD, but opposite to previous reports on mechanisms of dmPFC-rTMS in major depression. fMRI could prove useful in predicting the response to dmPFC-rTMS in OCD.

## INTRODUCTION

Obsessive-compulsive disorder (OCD) is a severely disabling psychiatric disorder with a lifetime prevalence of 1–3% (Abramowitz *et al*, 2009). OCD is characterized by intrusive, anxiety-provoking, ego-dystonic thoughts (obsessions), and associated repetitive behaviors (compulsions) (American Psychiatric Association, 1994). Of the OCD patients, 40–60% are refractory to conventional pharmacological and behavioral therapies (Pallanti *et al*, 2002). It is therefore crucial to develop novel therapies through a better understanding of the pathophysiology of OCD and the mechanisms of successful treatment.

Previous human and animal studies suggest that abnormalities in the cortico-striato-thalamic-cortical (CSTC) circuitry may be central to OCD pathophysiology (Menzies *et al*, 2008; Harrison *et al*, 2009; Ahmari *et al*, 2013; Admon *et al*, 2014). In healthy humans, specific CSTC loop circuits are important for self-regulation of affect, cognition, and behavior (Marsh *et al*, 2009; van den Heuvel *et al*, 2010; Lipsman *et al*, 2013). In OCD, these circuits display structural abnormalities relative to controls: volumetric gray matter reductions and reduced white matter integrity in the anterior cingulate cortex (Kühn *et al*, 2013), gray matter reductions in the orbitofrontal cortex (Rotge *et al*, 2010), and gray matter increases in thalamus and ventral striatum (Hou *et al*, 2013). On functional magnetic resonance imaging (fMRI), abnormal cortical-ventral striatal hyperconnectivity has been observed in OCD during a monetary incentive delay task (Beucke *et al*, 2012), during symptom provocation and during rest in many studies (Harrison *et al*, 2009; Cocchi *et al*, 2012; Figee *et al*, 2013; Anticevic *et al*, 2014). One study has shown the opposite, corticostriatal hypoconnectivity, in a group of unmedicated patients (Posner *et al*, 2014). In addition, altered anterior cingulate cortex metabolic activity has been observed in OCD on fluorodeoxyglucose-positron emission tomography and magnetic resonance spectroscopy (Perani *et al*, 1995; Ebert *et al*, 1997; Saxena *et al*, 2004, 2009; O'Neill *et al*, 2013). Taken together, these observations delineate a possible neuroanatomical substrate for OCD symptomatology.

Neuromodulation treatments offer a novel, anatomically targeted approach to refractory psychiatric conditions. Deep brain stimulation (DBS) has shown promising effects for OCD in recent studies (Greenberg *et al*, 2010); several CSTC targets have been explored, including the anterior limb of the internal capsule (Abelson *et al*, 2005), subthalamic nucleus (Mallet *et al*, 2008), and ventral striatum/nucleus accumbens (NAcc) (Denys *et al*, 2010; Figee *et al*, 2013). Regarding therapeutic mechanisms, a recent fMRI study found that NAcc-DBS normalized excessive functional connectivity between NAcc and dorsomedial and dorsolateral prefrontal cortex (dlPFC) in OCD patients; the degree of reduction correlated to the degree of symptomatic improvement (Figee *et al*, 2013).

Although DBS has shown promising effects in severe, refractory OCD cases, noninvasive forms of neuromodulation could be offered to a much wider range of patients. Repetitive transcranial magnetic stimulation (rTMS) could present a noninvasive alternative to DBS in OCD, if directed at a suitable stimulation target. rTMS to the dlPFC, although successful in major depression (O'Reardon *et al*, 2007; Berlim *et al*, 2014), has shown minimal clinical benefit over sham in double-blind trials for OCD (Alonso *et al*, 2001; Sachdev *et al*, 2007). However, medial prefrontal targets appear more promising: 1 Hz rTMS of the supplementary motor area (SMA) and pre-SMA has achieved substantial symptom improvement in case reports and randomized controlled trials (Mantovani *et al*, 2006, 2010a,b). Likewise, with transcranial direct current stimulation, cathodal but not anodal stimulation of the SMA has been reported to improve OCD symptoms (D'Urso *et al*, 2015).

A neighboring potential target is the dorsomedial prefrontal cortex (dmPFC), just anterior to the pre-SMA. Abnormally high resting state functional connectivity (Stern *et al*, 2012) and gray matter volume reductions in dmPFC (Radua *et al*, 2010) have been observed in OCD patients relative to controls. With NAcc-DBS (Figee *et al*, 2013), therapeutic efficacy correlated to reduction in excessive frontostriatal connectivity through the dmPFC regions. dmPFC-rTMS has not yet been studied in OCD. However, in major depressive disorder (MDD), a recent open-label study of 10 Hz dmPFC-rTMS for treatment-resistant MDD achieved ⩾50% symptom improvement in approximately half of the patients (Downar *et al*, 2014). Notably, therapeutic effects correlated to change in CSTC connectivity through the dmPFC on fMRI, as with NAcc-DBS in MDD (Salomons *et al*, 2014). These observations raise the possibility that dmPFC-rTMS might be able to engage a similar therapeutic mechanism through noninvasive means in OCD.

Here, we used resting-state fMRI to identify neural predictors and correlates of treatment response to 20–30 sessions of open-label, 10 Hz dmPFC-rTMS for refractory OCD. On the basis of our previous findings in MDD (Bakker *et al*, 2014; Downar *et al*, 2014; Salomons *et al*, 2014) and the aforementioned findings with NAcc-DBS in OCD (Figee *et al*, 2013), we hypothesized that pre-treatment resting-state dmPFC functional connectivity would predict response to dmPFC-rTMS in OCD, and that changes in resting-state dmPFC functional connectivity from pre- to post-treatment would correlate with the degree of symptomatic improvement.

## MATERIALS AND METHODS

### Subjects

Twenty OCD patients (4 male, mean age=37.3±15.5, range=21–63) participated in the study. OCD and comorbid Axis I and II disorders were diagnosed by a board-certified psychiatrist (authors JD, PG, PC, or BW) using DSM-IV criteria during a semi-structured clinical psychiatric evaluation incorporating the Mini International Neuropsychiatric Interview (MINI 6.0). Controls were screened using the same instrument. All patients reported at least one previous failed medication trial on clinical interview (ie not tolerated or clinically nonresponsive) (mean=5.7±4.1 trials) and 19 had failed at least one attempt at cognitive/behavioral intervention (ie did not complete the intervention or clinical nonresponsive). Mean illness duration was 24.2±15.2 years (range=8–54). No patients had hoarding symptomatology. Comorbidities included MDD (*n*=16), bipolar disorder (*n*=2), anorexia nervosa (*n*=5), bulimia nervosa (*n*=4), post-traumatic stress disorder (*n*=4), and Tourette's syndrome (*n*=1). No patients had a history of tics. Current medications included neuroleptic agents (*n*=12), selective serotonin reuptake inhibitors (*n*=8), serotonin-norepinephrine reuptake inhibitors (*n*=3), trazodone (*n*=2), lithium (*n*=1), and benzodiazepines (*n*=10); maximum daily benzodiazepine doses were 2 mg clonazepam, 4.5 mg bromazepam, and 0.75 mg alprazolam. Subjects were required to maintain a stable medication regimen for at least 4 weeks prior to and throughout rTMS treatment. All patients provided informed consent, and the study was approved by the University Health Network Research Ethics Board.

Forty healthy controls (17 male, mean age=34.88±11.76, range=18–66) were recruited to provide a comparator group for resting-state connectivity *post hoc* analyses. There was no difference in age and sex between the OCD and healthy control group (age *t*_58_=1.85, *p*=*n.s.*; sex χ^2^=2.97, *p*=*n.s.*). Healthy participants had no previous psychiatric diagnoses, no current psychiatric medication, and no current substance abuse or dependence, as verified by a screening interview by trained research staff. Healthy participants underwent MRI, but did not undergo a course of rTMS.

### Clinical Measures

Clinical measures were collected at baseline and 2 weeks post treatment. The primary outcome measure was the Yale-Brown Obsessive Compulsive Scale (YBOCS) (Goodman *et al*, 1989); in this study, treatment response was defined as ⩾50% improvement on the YBOCS. Secondary clinical measures included the 17-item Hamilton Rating Scale for Depression (HamD_17_), Beck Depression Index II (BDI-II), and Beck Anxiety Index (BAI). Additional clinical variables comprised the duration of illness, number of previous hospitalizations and outpatient treatment programs, number of previous medication trials, and current medication type and dose. Kernel density estimation of the distribution of clinical responses was performed in Stata13 (College Station, TX, USA).

### Intervention

dmPFC-rTMS was performed according a protocol we have previously reported for major depression (Downar *et al*, 2014), described in detail in the Supplementary Material. In summary, rTMS was delivered using the MagPro R30 system equipped with a Cool-DB80 coil (MagVenture, Farum, Denmark) and a Visor 2.0 neuronavigation system (Advanced NeuroTechnologies, Enschede, the Netherlands). Neuronavigation was performed for anatomical landmarking and co-registration of the brain to Talairach stereotaxic space, with the co-registered coil vertex coordinate (x0 y+60 z+60 in Talairach stereotaxic space), as performed in previously published work on dmPFC-rTMS (Bakker *et al*, 2014; Downar *et al*, 2014; Salomons *et al*, 2014). This location corresponds to approximately 25% of the distance from nasion to inion, slightly anterior to the location specified by previous authors targeting the pre-SMA for rTMS in OCD, which was at 35% of the nasion–inion distance (Mantovani *et al*, 2010a). Lateral coil orientation for preferential stimulation (Harmer *et al*, 2001; Terao *et al*, 2001) of the left then right dmPFC at 10 Hz, at 120% of the extensor halluces longus muscle resting motor threshold, with a duty cycle of 5 s on, 10 s off, for 60 trains (3000 pulses per hemisphere per session) for 20 daily sessions on weekdays, with non-remitters offered extension to 30 sessions.

### Neuroimaging Acquisition and Analysis

MRI acquisition parameters follow a protocol we have previously described in detail (Salomons *et al*, 2014), presented in full in the Supplementary Material. In summary, patients underwent MRI 1 week before and after rTMS treatment, to acquire a T1-weighted (0.94 × 0.94 × 1.5 mm) followed by a 10-min resting-state, eyes-closed T2* series (3.4 × 3.4 × 5 mm, TR=2 s). Healthy controls underwent a single session using identical parameters. Data preprocessing and analysis were performed in FSL (Jenkinson *et al*, 2012). Briefly, preprocessing included motion correction, slice-timing correction, spatial smoothing (6 mm FWHM Gaussian kernel), nuisance regression using 6 motion parameters and extracted white matter and cerebrospinal fluid mean times series, bandpass filter (0.009–0.09 Hz), and co-registration to the MNI-152 standard brain. Global signal regression was omitted from the pipeline to avoid the risk of introducing spurious anticorrelations in the results (Chai *et al*, 2012). Simple white matter and cerebrospinal fluid time series regression was performed as opposed to more complex techniques (eg, aCompCor) to maintain an identical pipeline to the atlas of resting-state functional connectivity used to obtain our seed as described below. FSL was then used to generate whole-brain maps of pre-treatment functional connectivity and of change in functional connectivity from pre-to post-treatment, to the dmPFC region-of-interest. The dmPFC region-of-interest (center of mass=MNI X 0, Y+38, Z+24) was defined from a resting-state connectivity-based atlas (Craddock *et al*, 2012), as previously for dmPFC-rTMS in MDD (Salomons *et al*, 2014). Other subcortical targets (the medial dorsal thalamus, three sites within ventral striatum, and subthalamic nucleus) were selected as exploratory seeds on the basis of their use as targets for DBS elsewhere in the literature, as reviewed in the Introduction; these seeds and the corresponding results are presented in the Supplementary Material.

The seed region-of-interest was co-registered to each subject, and its mean time series used as a regressor in a first-level analysis. To localize regions where pre-treatment functional connectivity correlated to treatment response, FSL's FLAME mixed effects model (Beckmann *et al*, 2003) was then applied for the group-level analysis using the responder/nonresponder status of each subject as a categorical, group-level regressor.

To localize regions where the *changes* in functional connectivity from pre- to post-treatment correlated to the degree of treatment response, a within-subjects, fixed-effects general linear model analysis was performed for each subject and the dmPFC seed region. For group-level analysis, these individual-subject beta-weighted change maps (ie, post- minus pre-rTMS) were then entered as first-level statistical maps into a between-subjects, mixed-effects linear regression analysis, using the responder/nonresponder status of each subject as a categorical, group-level independent variable.

Corrections for multiple comparisons were performed using Gaussian random field theory (Z>1.96, cluster significance *p*<0.05 corrected).

Parameter estimates for individual subjects' functional connectivity values (mean z-score) were then extracted for *post hoc* analysis. An 8-mm sphere centered at the peak cluster voxel (masked by the relevant cluster to ensure specificity) was registered from standard space to each individual subject using the transformation matrix from the original registration, extracting the mean z-score values from relevant connectivity maps. Healthy control parameter estimates were extracted in the same way.

## RESULTS

### Clinical Outcomes

Subjects completed a mean 21.3±4.1 sessions of 10 Hz dmPFC-rTMS (range=14–30). Treatment was well tolerated, with no serious or treatment-limiting adverse effects occurring. One subject discontinued treatment after 14 sessions owing to nonresponse, and was analyzed as a nonresponder using baseline measures.

**Figure 1:**
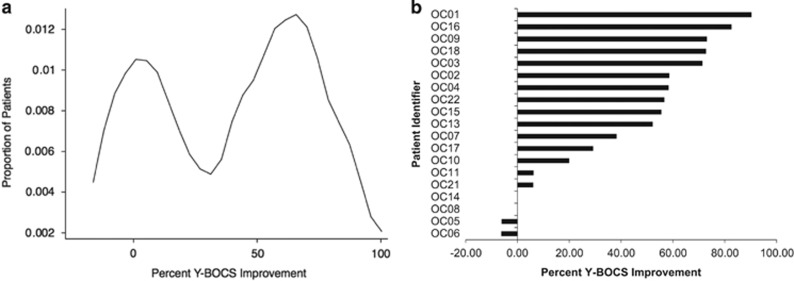
Probability distribution function (a) and ranked individual-patient plot (b) of treatment outcomes for dmPFC-rTMS in OCD. A bimodal distribution of treatment outcomes is evident, suggesting distinct responder and nonresponder subpopulations within the patient sample. Outcomes are calculated as percent improvement in YBOCS scores from pre- to post-treatment. One subject was a non-completer (14 rTMS sessions performed).

Across all subjects, baseline YBOCS scores significantly decreased from 30.5±4.3 to 18.4±10.8 (Wilcoxon rank-sum test, W_18_=3.41, *p*=0.007). However, kernel density estimation revealed a sharply bimodal response distribution, with distinct responder and nonresponder subpopulations (Figure 1); these subpopulations are therefore considered separately hereafter. Ten of 20 subjects met the response criterion of ⩾50% improvement. Among responders, YBOCS scores decreased 67.2%, from 29.3±4.6 to 9.6±3.9 (W_9_=2.81, *p*=0.005); among nonresponders, YBOCS scores decreased nonsignificantly by 11.4%, from 31.7±4.1 to 28.1±7.8 (W_8_=1.45, *p*=0.15). Of note, responders and nonresponders did not differ in YBOCS severity at baseline (29.3±4.6 vs 31.7±4.1; Mann–Whitney U_18_=1.21, *p*=0.22).

Regarding secondary measures, across all subjects, depression severity significantly improved on HamD_17_ from 17.7±7.7 to 9.9±7.3 (*t*_15_=3.08, *p*=0.008) and on BDI-II from 29.9±15.8 to 19.4±15.6 (*t*_18_=3.27, *p*=0.005). Again, outcomes were sharply dichotomous; YBOCS responders improved significantly on HamD_17_ from 15.8±5.8 to 5.8±4.0 (*t*_6_=3.64, *p*=0.01) and on BDI-II from 26.9±18.4 to 8.7±9.6 (*t*_9_=5.51, *p*=0.0006). YBOCS nonresponders showed no significant improvement on HamD_17,_ from 18.0±6.8 to 13.7±7.6 (*t*_9_=1.24, *p*=0.25), or on BDI-II, from 30.6±16.3 to 29.7±13.7 (*t*_8_=0.37, *p*=0.72). Anxiety symptomatology significantly improved on BAI from 28.7±15.0 to 15.9±12.4 (*t*_17_=4.01, *p*=0.0009) across all subjects, and from 29.8±18.3 to 10.4±10.9 (*t*_8_=5.11, *p*=0.0009) in YBOCS responders. YBOCS nonresponders showed nonsignificant improvement on BAI from 25.5±13.1 to 20.2±13.4 (*t*_8_=1.47, *p*=0.18). There was no difference in baseline severity for YBOCS responders vs YBOCS nonresponders on HamD17 (15.8±5.8 vs 18.0±6.8, *t*_15_=0.696, *p*=0.49), BDI-II (26.9±18.4 vs 30.6±16.3, *t*_18_=0.476, *p*=0.64), or BAI (29.8±18.3 vs 25.5±13.1, *t*_18_=0.602, *p*=0.55).

A Spearman's rank correlation coefficient was performed on baseline psychometric and clinical measures to determine whether any clinical factors predicted treatment outcome. No baseline factors (number of rTMS sessions, age, baseline severity, duration of illness, number of failed medications/treatments, comorbidities or current medication (antipsychotics, SSRI, or benzodiazepines), or baseline HamD_17_, BDI-II, or BAI) showed a significant correlation to treatment outcome, either before or after Bonferroni correction for multiple comparisons.

### fMRI Predictors of Treatment Response

Analysis of pre-treatment resting-state fMRI data revealed significant differences in baseline functional connectivity between responders and nonresponders for several exploratory seed regions-of-interest, including ventral rostral putamen to the dmPFC region (*p*=0.05) (see Supplementary Materials). The contrast of baseline functional connectivity between responders and nonresponders using the *a priori* dmPFC region showed trend toward higher connectivity among responders; however, the difference did not reach significance (*t*_15_=2.01, *p*=0.06).

### fMRI Correlates of Treatment Response

**Figure 2:**
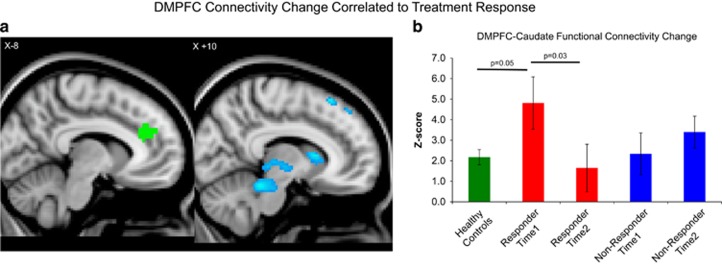
Reductions in cortical-striatal-thalamic connectivity correlate to improvements in OCD symptoms following dmPFC-rTMS. Bar graphs are intended to convey the absolute magnitudes of the parameter estimates in each group, as complementary information for the difference maps. (a) Regions of significant reduction in functional connectivity to the dmPFC seed (green) in rTMS-responders vs nonresponders are shown in blue. (b) Parameter estimates of functional connectivity between the dmPFC and caudate nucleus for healthy controls, responders, and nonresponders. Time 1, pre-treatment; Time 2, post-treatment.

Comparisons of pre-treatment and post-treatment resting-state fMRI data also revealed significant differences between responders and nonresponders in how functional connectivity to the seed regions *changed* over the course of treatment (Table 1). For the dmPFC seed, successful treatment response was associated with increased connectivity to the bilateral pre- and post-central gyrus and left precuneus, and decreased connectivity to the bilateral caudate nucleus, midbrain, thalamus, superior frontal gyrus, and right hippocampus (Table 1, Figure 2a). Responders showed significant decreases in dmPFC functional connectivity to the bilateral caudate (pre-treatment *z*=4.81±1.27, post-treatment *z*=1.65±1.16, *t*_8_=2.61, *p*=0.03), and thalamus (pre-treatment *z*=0.64±1.24, post-treatment *z*=−3.37±1.01, *t*_8_=2.57, *p*=0.03). Conversely, nonresponders showed significant *increases* in functional connectivity from dmPFC to the hippocampus (pre-treatment *z*=−3.19±0.83, post-treatment *z*=−0.86±0.88, *t*_6_=3.24, *p*=0.01) and midbrain (pre-treatment *z*=−3.71±1.00, post-treatment *z*=−1.20±0.86, *t*_6_=2.50, *p*=0.04). Again, across all subjects, the percent YBOCS improvement correlated significantly to the degree of reduction in dmPFC functional connectivity to the caudate (*r*_17_=−0.56, *p*=0.02) and hippocampus(*r*_15_=−0.58, *p*=0.02).

Compared with healthy controls, responders' dmPFC-caudate connectivity was significantly higher at baseline (control *z*=3.04±0.85, *t*_47_=2.05, *p*=0.05) and nonsignificantly higher compared with nonresponders (responder *z*=4.81±1.27; nonresponder *z*=2.34±1.16, *t*_15_=2.01, *p*=0.06) (Figure 2b).

Remaining analyses performed on exploratory seeds can be found in the Supplementary Materials.

## DISCUSSION

To our knowledge, this is the first case series using fMRI to identify neural predictors and correlates of response to any form of noninvasive brain stimulation in OCD. Previous studies of rTMS in OCD have encountered little therapeutic benefit with stimulation of lateral prefrontal targets such as the dlPFC (Alonso *et al*, 2001; Prasko *et al*, 2006; Sachdev *et al*, 2007; Sarkhel *et al*, 2010), but somewhat more success with medial prefrontal targets such as the SMA or pre-SMA (Mantovani *et al*, 2006, 2010a,b; Kumar and Chadda, 2011). The results of the present study are congruent with this latter literature in suggesting that rTMS of a slightly more anterior medial prefrontal target, the dmPFC, can also yield substantial symptom reduction in a proportion of OCD cases, even when multiple previous medication trials have failed. However, as previously observed in patients with depression (Downar *et al*, 2014), dmPFC-rTMS outcomes appear sharply bimodal in OCD.

In keeping with our first hypothesis, responders showed significant differences from nonresponders in baseline dmPFC-striatal resting-state functional connectivity. Furthermore, in keeping with our second hypothesis, the amount of symptomatic improvement correlated to the degree of reduction in functional connectivity through a very similar frontal-striatal-thalamic-subthalamic circuit connecting the dmPFC to VS, caudate nucleus, thalamus, and midbrain. In relation to healthy controls, responders initially showed hyperconnectivity from dmPFC to caudate before treatment, and this hyperconnectivity normalized following treatment; no such pattern was evident in nonresponders.

These observations are remarkably consistent with the recently reported results of an fMRI study of therapeutic mechanisms in OCD, using ventral striatum-DBS rather than rTMS of the dmPFC (Figee *et al*, 2013). In that study, OCD patients also showed baseline functional hyperconnectivity from ventral striatum to a dmPFC region just immediately anterior to the region identified in Figure 2 of the present study. DBS reduced functional connectivity from ventral striatum to dmPFC, and the degree of reduction correlated well with the amount of symptomatic improvement. The results of the present study suggest that similar therapeutic effects in OCD might also be achieved via noninvasive stimulation of the more superficial dmPFC, via a similar mechanism. Notably, the circuit identified in the present study also includes nodes in lateral orbitofrontal cortex, ventral striatum, medial thalamus, and subthalamic nucleus, each of which has shown promise as a therapeutic target for either DBS (Abelson *et al*, 2005; Mallet *et al*, 2008; Denys *et al*, 2010; Lipsman *et al*, 2013), or in the case of lateral orbitofrontal cortex, rTMS (Ruffini *et al*, 2009).

More generally, the observations of the present study support the growing body of evidence that OCD pathophysiology may arise from functional hyperconnectivity through specific cortico-striatal loop circuits projecting from ventral striatum to the medial PFC (Harrison *et al*, 2009; Sakai *et al*, 2011; Beucke *et al*, 2013). Recent studies, in larger patient samples, have even suggested that different OCD symptom dimensions may map on to anatomically distinct cortico-striatal pathways (Harrison *et al*, 2013). Animal studies also suggest that the dynamics of the hyperactivation may be important. For example, a recent study showed that repeated exposures to brief optogenetic hyperactivation of an orbitofrontal cortex-ventral striatal circuit in mice led to progressively increased compulsive grooming behaviors that were reversible by fluoxetine, a standard pharmacotherapy for OCD (Ahmari *et al*, 2013). These more nuanced techniques could lead to a better understanding of the variability of response to current neuromodulation therapies in OCD.

We also note that the fMRI predictors and correlates of successful 10 Hz dmPFC-rTMS for OCD appear *opposite* to those we have previously reported for the same intervention in major depression (Salomons *et al*, 2014). In the MDD patients, *low* baseline connectivity from dmPFC to the putamen and thalamus predicted better response to treatment, and the degree of *increase* in frontal-striatal-thalamic connectivity correlated to symptomatic improvement. The results of the present study suggest that dmPFC-rTMS may exert therapeutic effects via a similar CSTC pathway in both MDD and OCD, but via opposite mechanisms (ie, via reduction of a pathologically high baseline CTSC connectivity in OCD, rather than via strengthening of a pathologically low baseline CTSC connectivity in MDD). These seemingly contrary findings could potentially be reconciled if dmPFC-rTMS exerts its therapeutic effects on OCD and MDD not directly but indirectly, by relieving intrusive thoughts, as suggested by a recent study (Carew *et al*, 2013). However, this issue will require further study.

One important difference in technique between the present study and previous studies of rTMS in OCD targeting the medial wall is that the previous studies used 1 Hz rather than 10 Hz stimulation. Classically, 1 Hz stimulation is considered inhibitory, and 10 Hz stimulation excitatory (Hallett, 2007); suppression of overactive regions in SMA and pre-SMA provided a rationale for using 1 Hz stimulation in these previous studies (Mantovani *et al*, 2006, 2010a,b). Yet it is increasingly recognized that the effects of many rTMS protocols, including both 1 and 10 Hz stimulation, can be quite heterogeneous: a substantial proportion of individuals show ‘paradoxical' excitatory responses to 1 Hz stimulation or inhibitory responses to high-frequency stimulation, both on motor evoked potentials (Maeda *et al*, 2000; Hamada *et al*, 2013) and on resting-state fMRI (Eldaief *et al*, 2011). In the present study, as well as in our previous study of dmPFC-rTMS in MDD, the effects of 10 Hz stimulation on cortical-striatal-thalamic activity were in fact quite variable across individuals, with ‘paradoxical' reductions in functional connectivity appearing in up to 40% of MDD patients in the previous study and in more than half of the OCD patients in the present study (Figure 3). Thus, it is possible that 1 Hz SMA-rTMS and 10 Hz dmPFC-rTMS are achieving successful OCD treatment outcomes via similar mechanisms, notwithstanding the differences in technique. Alternatively, individual patients may require different sites or patterns of stimulation to achieve a therapeutic effect, as has been reported for dlPFC-rTMS in depression (Speer *et al*, 2009). The question of whether 1 Hz SMA-rTMS and 10 Hz dmPFC-rTMS treat similar or different subpopulations of OCD patients will be an important topic for future study. In either case, resting-state fMRI is likely to have an important role both in stratifying patients and in clarifying therapeutic mechanisms.

One limitation of the present study is use of an open-label design, leaving open the possibility that the observed symptomatic improvements were due to non-specific or placebo effects. However, it should be noted that in the setting of refractory OCD, previously reported effects of sham rTMS are relatively minimal, ranging from 1% to ~20% improvement in YBOCS scores; indeed, even active stimulation of lateral targets such as dlPFC has consistently achieved less than 25% YBOCS improvement across several independent studies (Alonso *et al*, 2001; Sachdev *et al*, 2007; Mantovani *et al*, 2010a; Sarkhel *et al*, 2010). Thus, it is unlikely that placebo effects can fully account for the present observations of a ~40% overall improvement in the present study, a bimodal outcome distribution, distinct patterns of functional connectivity through the dmPFC-ventral striatal target circuit in responders vs nonresponders (who were not otherwise distinct on clinical measures), distinct patterns of change in this circuit in responders and nonresponders, and the concordance of these results with the previous findings of an independent study using DBS rather than rTMS. Nonetheless, replication of the present findings under sham-controlled conditions would be an important next step in this line of research.

Another potential criticism of the current study relates to the small sample size. Although the sample size here is comparable with or larger than that in other dmPFC rTMS-MRI and DBS-MRI studies (Figee *et al*, 2013; Salomons *et al*, 2014), open-label and randomized control rTMS treatment studies for OCD (Mantovani *et al*, 2006, 2010a,b) and neuroimaging studies for OCD (Admon *et al*, 2012), it is nonetheless underpowered to capture the full heterogeneity of OCD symptomatology, comorbidity, and treatment types. Hence, the present observations are as yet insufficient to properly address the question as to whether dmPFC-rTMS response could also be predicted on the basis of clinical features such as OCD subtype (eg, hoarding vs checking), presence or absence of comorbid symptoms (eg, major depression, tics), or adjunctive treatment types (eg, use of SSRIs or neuroleptics). They also cannot yet address the question of whether dmPFC-rTMS might selectively improve some OCD symptom dimensions but not others, as might be suspected if different symptom clusters map reliably on to distinct neural substrates. Resolution of such issues must await a larger sample of patients.

In summary, 10 Hz dmPFC-rTMS may offer a promising, noninvasive therapeutic option for medication-refractory OCD, achieving ⩾50% reductions in YBOCS scores in 50% of the patients in the sample. In agreement with recent findings with ventral striatal DBS for OCD, rTMS may be most effective in patients with greater hyperconnectivity between dmPFC and ventral striatum on resting-state fMRI. Also in agreement with DBS findings, therapeutic effects of dmPFC-rTMS correlated with reductions in dmPFC-ventral striatum functional connectivity. A randomized controlled trial incorporating a sham rTMS arm would be a logical next step in evaluating dmPFC-rTMS as a noninvasive alternative to DBS in medication-refractory OCD. Future studies of rTMS in OCD may also benefit from using fMRI to characterize cortico-striatal connectivity prior to treatment, in order to predict response and/or tailor the stimulation target and parameters in individual patients (Fox *et al*, 2013). Properly optimized, rTMS could evolve into a potent, novel treatment option for patients faced with this challenging and crippling illness.

## Funding and Disclosure

Dr Downar has received research support from the Canadian Institutes of Health Research, the National Institutes of Health, the Klarman Family Foundation, the Buchan Family Foundation, and the Toronto General and Western Hospital Foundation, as well as a travel stipend from Lundbeck and in-kind equipment support for an investigator-initiated study from MagVenture. Dr Giacobbe is a consultant for St Jude Medical and has received personal fees from Eli Lilly Canada, Bristol-Myers Squibb, AstraZeneca, and Pfizer. He has also received research support from the Canadian Institutes of Health Research, Michael J. Fox Foundation for Parkinson's Research, the Brain and Behavior Research Foundation (formerly National Alliance for Research on Schizophrenia and Depression), and the National Institutes of Health. Dr Woodside has received research support from the Ontario Mental Health Foundation, the Price Foundation, the Klarman Foundation, the Canadian Institute for Health Research, and the National Institute for Mental Health. Ms. Dunlop has received research support from the Canadian Institutes for Health Research Vanier Scholarship. Drs Colton and Olmsted report no financial relationships with commercial interests.

## Figures and Tables

**Table 1:** Brain Regions Where the Pre-to-Post Treatment Change in Functional Connectivity to the dmPFC Differed Significantly Between rTMS Responders and Nonresponders

			**MNI coordinate**			
**Seed**	**Region**	**Brodmann area**	**X**	**Y**	**Z**	**Peak Z score**
**dmPFC**						
*FC increase in Resp >Nonresp*						
L	Precuneus	7	−6	−48	62	3.93
L	Postcentral gyrus	7	−4	−44	70	4.23
L	Precentral gyrus	6	−16	−30	54	3.83
R	Precentral gyrus	6	4	−20	60	2.87
R	Postcentral gyrus	7	14	−36	48	2.74
						
*FC reduction in Resp >Nonresp*						
L	Superior frontal gyrus	6	−10	14	64	4.84
R	Superior frontal gyrus	6	12	26	58	3.33
L	Caudate nucleus		−8	14	6	2.72
R	Caudate nucleus		6	14	8	3.40
R	Thalamus		22	−4	8	3.07
L	Thalamus/Putamen		−6	−14	2	3.02
R	Hippocampus		30	−22	−8	3.39
B	Dorsal midbrain		−6	−28	−10	2.57
B	Ventral midbrain		−2	−16	−16	2.61

Abbreviations: dmPFC, dorsomedial prefrontal cortex; FC, functional connectivity; MNI, Montreal Neurological Institute; Nonresp, nonresponder; Resp, responder.

All activations are Gaussian random field theory corrected for multiple comparisons at a cluster threshold *p*<0.05.

## References

[bib1] Abelson JL, Curtis GC, Sagher O, Albucher RC, Harrigan M, Taylor SF et al (2005). Deep brain stimulation for refractory obsessive-compulsive disorder. Biol Psychiatry 57: 510–516. 15737666 10.1016/j.biopsych.2004.11.042

[bib2] Abramowitz JS, Taylor S, McKay D (2009). Obsessive-compulsive disorder. Lancet 374: 491–499. 19665647 10.1016/S0140-6736(09)60240-3

[bib3] Admon R, Bleich-Cohen M, Weizmant R, Poyurovsky M, Faragian S, Hendler T (2012). Functional and structural neural indices of risk aversion in obsessive-compulsive disorder (OCD). Psychiatry Res 203: 207–213. 22959813 10.1016/j.pscychresns.2012.02.002

[bib4] Admon R, Nickerson LD, Dillon DG, Holmes AJ, Bogdan R, Kumar P et al (2014). Dissociable cortico-striatal connectivity abnormalities in major depression in response to monetary gains and penalties. Psychol Med 45: 121–131. 25055809 10.1017/S0033291714001123 PMC4233014

[bib5] Ahmari SE, Spellman T, Douglass NL, Kheirbek Ma, Simpson HB, Deisseroth K et al (2013). Repeated cortico-striatal stimulation generates persistent OCD-like behavior. Science 340: 1234–1239. 23744948 10.1126/science.1234733 PMC3954809

[bib6] Alonso P, Pujol J, Cardoner N, Benlloch L, Deus J, Menchón JM et al (2001). Right prefrontal repetitive transcranial magnetic stimulation in obsessive-compulsive disorder: a double-blind, placebo-controlled study. Am J Psychiatry 158: 1143–1145. 11431238 10.1176/appi.ajp.158.7.1143

[bib7] American Psychiatric Association (1994) Diagnostic and Statistical Manual of Mental Disorders, 4th ed. American Psychiatric Association: Washington (DC), 866p.

[bib8] Anticevic A, Hu S, Zhang S, Savic A, Billingslea E, Wasylink S et al (2014). Global resting-state functional magnetic resonance imaging analysis identifies frontal cortex, striatal, and cerebellar dysconnectivity in obsessive-compulsive disorder. Biol Psychiatry 75: 595–605. 24314349 10.1016/j.biopsych.2013.10.021 PMC3969771

[bib9] Bakker N, Shahab S, Giacobbe P, Blumberger DM, Daskalakis ZJ, Kennedy SH et al (2015). rTMS of the dorsomedial prefrontal cortex for major depression: safety, tolerability, effectiveness, and outcome predictors for 10 Hz versus intermittent theta-burst stimulation. Brain Stimul 8: 1–22. 25465290 10.1016/j.brs.2014.11.002

[bib10] Beckmann CF, Jenkinson M, Smith SM (2003). General multilevel linear modeling for group analysis in FMRI. Neuroimage 20: 1052–1063. 14568475 10.1016/S1053-8119(03)00435-X

[bib11] Berlim MT, van den Eynde F, Tovar-Perdomo S, Daskalakis ZJ (2014). Response, remission and drop-out rates following high-frequency repetitive transcranial magnetic stimulation (rTMS) for treating major depression: a systematic review and meta-analysis of randomized, double-blind and sham-controlled trials. Psychol Med 44: 225–239. 23507264 10.1017/S0033291713000512

[bib12] Beucke JC, Kaufmann C, Linnman C, Gruetzmann R, Endrass T, Deckersbach T et al (2012). Altered cingulostriatal coupling in obsessive-compulsive disorder. Brain Connect 2: 191–202. 22823561 10.1089/brain.2012.0078

[bib13] Beucke JC, Sepulcre J, Talukdar T, Linnman C, Zschenderlein K, Endrass T et al (2013). Abnormally high degree connectivity of orbitofrontal cortex in obsessive-compulsive disorder. JAMA Psychiatry 70: 619–629. 23740050 10.1001/jamapsychiatry.2013.173

[bib14] Carew CL, Milne AM, Tatham EL, MacQueen GM, Hall GB (2013). Neural systems underlying thought suppression in young women with, and at-risk, for depression. Behav Brain Res 257: 13–24. 24055881 10.1016/j.bbr.2013.09.016

[bib15] Chai XJ, Castañán AN, Öngür D, Whitfield-Gabrieli S (2012). Anticorrelations in resting state networks without global signal regression. Neuroimage 59: 1420–1428. 21889994 10.1016/j.neuroimage.2011.08.048 PMC3230748

[bib16] Cocchi L, Harrison BJ, Pujol J, Harding IH, Fornito A, Pantelis C et al (2012). Functional alterations of large-scale brain networks related to cognitive control in obsessive-compulsive disorder. Hum Brain Mapp 33: 1089–1106. 21612005 10.1002/hbm.21270 PMC6870338

[bib17] Craddock RC, James GA, Holtzheimer PE, Hu XP, Mayberg HS (2012). A whole brain fMRI atlas generated via spatially constrained spectral clustering. Hum Brain Mapp 33: 1914–1928. 21769991 10.1002/hbm.21333 PMC3838923

[bib18] Denys D, Mantione M, Figee M, van den Munckhof P, Koerselman F, Westenberg H et al (2010). Deep brain stimulation of the nucleus accumbens for treatment-refractory obsessive-compulsive disorder. Arch Gen Psychiatry 67: 1061–1068. 20921122 10.1001/archgenpsychiatry.2010.122

[bib19] Downar J, Geraci J, Salomons TV, Dunlop K, Wheeler S, McAndrews MP et al (2014). Anhedonia and reward-circuit connectivity distinguish nonresponders from responders to dorsomedial prefrontal repetitive transcranial magnetic stimulation in major depression. Biol Psychiatry 76: 176–185. 24388670 10.1016/j.biopsych.2013.10.026

[bib20] D'Urso G, Brunoni AR, Anastasia A, Micillo M, de Bartolomeis A, Mantovani A (2015). Polarity-dependent effects of transcranial direct current stimulation in obsessive-compulsive disorder. Neurocase 14: 1–5. 10.1080/13554794.2015.1045522 25971992

[bib21] Ebert D, Speck O, König A, Berger M, Hennig J, Hohagen F (1997). 1H-magnetic resonance spectroscopy in obsessive-compulsive disorder: evidence for neuronal loss in the cingulate gyrus and the right striatum. Psychiatry Res 74: 173–176. 9255862 10.1016/s0925-4927(97)00016-4

[bib22] Eldaief MC, Halko MA, Buckner RL, Pascual-Leone A (2011). Transcranial magnetic stimulation modulates the brain's intrinsic activity in a frequency-dependent manner. Proc Natl Acad Sci USA 108: 21229–21234. 22160708 10.1073/pnas.1113103109 PMC3248528

[bib23] Figee M, Luigjes J, Smolders R, Valencia-Alfonso C-E, van Wingen G, de Kwaasteniet B et al (2013). Deep brain stimulation restores frontostriatal network activity in obsessive-compulsive disorder. Nat Neurosci 16: 386–387. 23434914 10.1038/nn.3344

[bib24] Fox MD, Liu H, Pascual-Leone A (2013). Identification of reproducible individualized targets for treatment of depression with TMS based on intrinsic connectivity. Neuroimage 66: 151–160. 23142067 10.1016/j.neuroimage.2012.10.082 PMC3594474

[bib25] Goodman WK, Price LH, Rasmussen SA, Mazure C, Fleischmann RL, Hill CL et al (1989). The Yale-Brown Obsessive Compulsive Scale. I. Development, use, and reliability. Arch Gen Psychiatry 46: 1006–1011. 2684084 10.1001/archpsyc.1989.01810110048007

[bib26] Greenberg BD, Rauch SL, Haber SN (2010). Invasive circuitry-based neurotherapeutics: stereotactic ablation and deep brain stimulation for OCD. Neuropsychopharmacology 35: 317–336. 19759530 10.1038/npp.2009.128 PMC3055421

[bib27] Hallett M (2007). Transcranial magnetic stimulation: a primer. Neuron 55: 187–199. 17640522 10.1016/j.neuron.2007.06.026

[bib28] Hamada M, Murase N, Hasan A, Balaratnam M, Rothwell JC (2013). The role of interneuron networks in driving human motor cortical plasticity. Cereb Cortex 23: 1593–1605. 22661405 10.1093/cercor/bhs147

[bib29] Harmer CJ, Thilo KV, Rothwell JC, Goodwin GM (2001). Transcranial magnetic stimulation of medial-frontal cortex impairs the processing of angry facial expressions. Nat Neurosci 4: 17–18. 11135640 10.1038/82854

[bib30] Harrison BJ, Pujol J, Cardoner N, Deus J, Alonso P, Lopez-Sola M et al (2013). Brain corticostriatal systems and the major clinical symptom dimensions of obsessive-compulsive disorder. Biol Psychiatry 73: 321–328. 23200527 10.1016/j.biopsych.2012.10.006

[bib31] Harrison BJ, Soriano-Mas C, Pujol J, Ortiz H, Lopez-Sola M, Hernandez-Ribas R et al (2009). Altered corticostriatal functional connectivity in obsessive-compulsive disorder. Arch Gen Psychiatry 66: 1189–1200. 19884607 10.1001/archgenpsychiatry.2009.152

[bib32] Hou J, Song L, Zhang W, Wu W, Wang J, Zhou D et al (2013). Morphologic and functional connectivity alterations of corticostriatal and default mode network in treatment-naïve patients with obsessive-compulsive disorder. PLoS One 8: e83931. 24358320 10.1371/journal.pone.0083931 PMC3865285

[bib33] Jenkinson M, Beckmann CF, Behrens TE, Woolrich MW, Smith SM (2012). FSL. Neuroimage 62: 782–790. 21979382 10.1016/j.neuroimage.2011.09.015

[bib34] Kumar N, Chadda RK (2011). Augmentation effect of repetitive transcranial magnetic stimulation over the supplementary motor cortex in treatment refractory patients with obsessive compulsive disorder. Indian J Psychiatry 53: 340–342. 22303044 10.4103/0019-5545.91909 PMC3267347

[bib35] Kühn S, Kaufmann C, Simon D, Endrass T, Gallinat J, Kathmann N (2013). Reduced thickness of anterior cingulate cortex in obsessive-compulsive disorder. Cortex 49: 2178–2185. 23067727 10.1016/j.cortex.2012.09.001

[bib36] Lipsman N, Giacobbe P, Lozano AM (2013). Deep brain stimulation in obsessive-compulsive disorder: neurocircuitry and clinical experience. Handb Clin Neurol 116: 245–250. 24112898 10.1016/B978-0-444-53497-2.00019-X

[bib37] Maeda F, Keenan JP, Tormos JM, Topka H, Pascual-Leone A (2000). Interindividual variability of the modulatory effects of repetitive transcranial magnetic stimulation on cortical excitability. Exp Brain Res 133: 425–430. 10985677 10.1007/s002210000432

[bib38] Mallet L, Polosan M, Jaafari N, Baup N, Welter M-L, Fontaine D et al (2008). Subthalamic nucleus stimulation in severe obsessive-compulsive disorder. N Engl J Med 359: 2121–2134. 19005196 10.1056/NEJMoa0708514

[bib39] Mantovani A, Lisanby SH, Pieraccini F, Ulivelli M, Castrogiovanni P, Rossi S (2006). Repetitive transcranial magnetic stimulation (rTMS) in the treatment of obsessive-compulsive disorder (OCD) and Tourette's syndrome (TS). Int J Neuropsychopharmacol 9: 95–100. 15982444 10.1017/S1461145705005729

[bib40] Mantovani A, Simpson HB, Fallon BA, Rossi S, Lisanby SH (2010a). Randomized sham-controlled trial of repetitive transcranial magnetic stimulation in treatment-resistant obsessive-compulsive disorder. Int J Neuropsychopharmacol 13: 217–227. 19691873 10.1017/S1461145709990435

[bib41] Mantovani A, Westin G, Hirsch J, Lisanby SH (2010b). Functional magnetic resonance imaging guided transcranial magnetic stimulation in obsessive-compulsive disorder. Biol Psychiatry 67: e39–e40. 19793582 10.1016/j.biopsych.2009.08.009

[bib42] Marsh R, Maia TV, Peterson BS (2009). Functional disturbances within frontostriatal circuits across multiple childhood psychopathologies. Am J Psychiatry 166: 664–674. 19448188 10.1176/appi.ajp.2009.08091354 PMC2734479

[bib43] Menzies L, Chamberlain S, Laird A, Thelen S, Sahakian BJ, Bullmore E (2008). Integrating evidence from neuroimaging and neuropsychological studies of obsessive-compulsive disorder: the orbitofronto-striatal model revisisted. Neurosci Biobehav Rev 32: 525–549. 18061263 10.1016/j.neubiorev.2007.09.005 PMC2889493

[bib44] O'Neill J, Gorbis E, Feusner JD, Yip JC, Chang S, Maidment KM et al (2013). Effects of intensive cognitive-behavioral therapy on cingulate neurochemistry in obsessive-compulsive disorder. J Psychiatr Res 47: 494–504. 23290560 10.1016/j.jpsychires.2012.11.010 PMC3672238

[bib45] O'Reardon JP, Solvason HB, Janicak PG, Sampson S, Isenberg KE, Nahas Z et al (2007). Efficacy and safety of transcranial magnetic stimulation in the acute treatment of major depression: a multisite randomized controlled trial. Biol Psychiatry 62: 1208–1216. 17573044 10.1016/j.biopsych.2007.01.018

[bib46] Pallanti S, Hollander E, Bienstock C, Koran L, Leckman J, Marazziti D et al (2002). Treatment non-response in OCD: methodological issues and operational definitions. Int J Neuropsychopharmacol 5: 181–191. 12135542 10.1017/S1461145702002900

[bib47] Perani D, Colombo C, Bressi S, Bonfanti A, Grassi F, Scarone S et al (1995). [18F]FDG PET study in obsessive-compulsive disorder. A clinical/metabolic correlation study after treatment. Br J Psychiatry 166: 244–250. 7728370 10.1192/bjp.166.2.244

[bib48] Posner J, Marsh R, Maia TV, Peterson BS, Gruber A, Simpson HB (2014). Reduced functional connectivity within the limbic cortico-striato-thalamo-cortical loop in unmedicated adults with obsessive-compulsive disorder. Hum Brain Mapp 35: 2852–2860. 24123377 10.1002/hbm.22371 PMC4142493

[bib49] Prasko J, Paskova B, Zalesky R, Novak T, Kopecek M, Bares M et al (2006). The effect of repetitive transcranial magnetic stimulation (rTMS) on symptoms in obsessive compulsive disorder. A randomized, double blind, sham controlled study. Neuro Endocrinol Lett 27: 327–332. 16816829

[bib50] Radua J, van den Heuvel OA, Surguladze S, Mataix-Cols D (2010). Meta-analytical comparison of voxel-based morphometry studies in obsessive-compulsive disorder vs other anxiety disorders. Arch Gen Psychiatry 67: 701–711. 20603451 10.1001/archgenpsychiatry.2010.70

[bib51] Rotge J-Y, Langbour N, Guehl D, Bioulac B, Jaafari N, Allard M et al (2010). Gray matter alterations in obsessive-compulsive disorder: an anatomic likelihood estimation meta-analysis. Neuropsychopharmacology 35: 686–691. 19890260 10.1038/npp.2009.175 PMC3055616

[bib52] Ruffini C, Locatelli M, Lucca A, Benedetti F, Insacco C, Smeraldi E (2009). Augmentation effect of repetitive transcranial magnetic stimulation over the orbitofrontal cortex in drug-resistant obsessive-compulsive disorder patients: a controlled investigation. Prim Care Companion J Clin Psychiatry 11: 226–230. 19956460 10.4088/PCC.08m00663 PMC2781034

[bib53] Sachdev PS, Loo CK, Mitchell PB, McFarquhar TF, Malhi GS (2007). Repetitive transcranial magnetic stimulation for the treatment of obsessive compulsive disorder: a double-blind controlled investigation. Psychol Med 37: 1645–1649. 17655805 10.1017/S0033291707001092

[bib54] Sakai Y, Narumoto J, Nishida S, Nakamae T, Yamada K, Nishimura T et al (2011). Corticostriatal functional connectivity in non-medicated patients with obsessive-compulsive disorder. Eur Psychiatry 26: 463–469. 21067900 10.1016/j.eurpsy.2010.09.005

[bib55] Salomons TV, Dunlop K, Kennedy SH, Flint A, Geraci J, Giacobbe P et al (2014). Resting-state cortico-thalamic-striatal connectivity predicts response to dorsomedial prefrontal rTMS in major depressive disorder. Neuropsychopharmacology 39: 488–498. 24150516 10.1038/npp.2013.222 PMC3870791

[bib56] Sarkhel S, Sinha VK, Praharaj SK (2010). Adjunctive high-frequency right prefrontal repetitive transcranial magnetic stimulation (rTMS) was not effective in obsessive-compulsive disorder but improved secondary depression. J Anxiety Disord 24: 535–539. 20392594 10.1016/j.janxdis.2010.03.011

[bib57] Saxena S, Brody AL, Maidment KM, Smith EC, Zohrabi N, Katz E et al (2004). Cerebral glucose metabolism in obsessive-compulsive hoarding. Am J Psychiatry 161: 1038–1048. 15169692 10.1176/appi.ajp.161.6.1038

[bib58] Saxena S, Gorbis E, O'Neill J, Baker SK, Mandelkern MA, Maidment KM et al (2009). Rapid effects of brief intensive cognitive-behavioral therapy on brain glucose metabolism in obsessive-compulsive disorder. Mol Psychiatry 14: 197–205. 18180761 10.1038/sj.mp.4002134 PMC2893580

[bib59] Speer AM, Benson BE, Kimbrell TK, Wassermann EM, Willis MW, Herscovitch P et al (2009). Opposite effects of high and low frequency rTMS on mood in depressed patients: relationship to baseline cerebral activity on PET. J Affect Disord 115: 386–394. 19027962 10.1016/j.jad.2008.10.006 PMC2779113

[bib60] Stern ER, Fitzgerald KD, Welsh RC, Abelson JL, Taylor SF (2012). Resting-state functional connectivity between fronto-parietal and default mode networks in obsessive-compulsive disorder. PLoS One 7: e36356. 22570705 10.1371/journal.pone.0036356 PMC3343054

[bib61] Terao Y, Ugawa Y, Enomoto H, Furubayashi T, Shiio Y, Machii K et al (2001). Hemispheric lateralization in the cortical motor preparation for human vocalization. J Neurosci 21: 1600–1609. 11222650 10.1523/JNEUROSCI.21-05-01600.2001 PMC6762942

[bib62] van den Heuvel OA, van der Werf YD, Verhoef KMW, de Wit SJ, Berendse HW, Wolters EC et al (2010). Frontal-striatal abnormalities underlying behaviours in the compulsive-impulsive spectrum. J Neurol Sci 289: 55–59. 19729172 10.1016/j.jns.2009.08.043

