# Supplementary material for: Reductions in Cortico-Striatal Hyperconnectivity Accompany Successful Treatment of Obsessive-Compulsive Disorder with Dorsomedial Prefrontal rTMS
Source: Neuropsychopharmacology. 2015 Oct 28;41(5):1395–403. doi: 10.1038/npp.2015.292 (PMC4793124; doi:10.1038/npp.2015.292)
Supplement: Supplementary Information [file npp2015292x1.doc]

**SUPPLEMENTARY MATERIALS**

***Reductions in cortico-striatal hyperconnectivity accompany successful treatment of obsessive-compulsive disorder with dorsomedial prefrontal rTMS.***

Katharine Dunlop^1,2^, Blake Woodside^1,3,5,6^, Marion Olmsted^5,6^, Patricia Colton^3,5,6^, Peter Giacobbe^2,3,5^, Jonathan Downar^1,2,3,4,5^

^1^Institute of Medical Sciences, University of Toronto

^2^MRI-Guided rTMS Clinic, University Health Network

^3^Department of Psychiatry, University Health Network, Toronto, Canada

^4^Toronto Western Research Institute, University Health Network, Toronto, Canada

^5^Department of Psychiatry, University of Toronto

^6^Eating Disorders Program, University Health Network, Toronto, Canada

*Corresponding author:*

Jonathan Downar, MD PhD FRCPC

MRI-Guided rTMS Clinic, University Health Network

399 Bathurst Street 7M-415

Toronto, Ontario, Canada M5T 2S8

T 416 603 5667 F 416 603 5292 jonathan.downar@uhn.ca

**rTMS Procedures**

rTMS was delivered under MRI guidance using the MagPro R30 system equipped with a Cool-DB80 coil (MagVenture, Farum, Denmark) and the Visor 2.0 neuronavigation system (Advanced Neuro Technologies, Enschede, Netherlands). The Visor software was used for anatomical landmarking and coregistration of the brain into the standard stereotaxic space of Talairach and Tournoux, followed by segmentation of T1 images into scalp and brain compartments, rendering of 3D surfaces for the scalp and brain, and coregistration of these surfaces to the patient’s head for coil placement during treatment. As in our previous studies of dmPFC-rTMS(Salomon*s et* al, 2014), we selected a dmPFC target region at the stereotaxic coordinate (X 0 Y+30 Z +30) for stimulation and the scalp point closest to the coordinate (X 0 Y+60 Z+60) was used as the focal point for coil placement during treatment. This point corresponds to approximately 25% of the distance from nasion to inion (slightly anterior to Fz according to the international 10-20 EEG electrode positioning system).

Motor thresholding methods for dmPFC-rTMS followed a protocol we have previously reported(Salomon*s et* al, 2014). The coil vertex was placed over the interhemispheric fissure, immediately anterior to the central sulcus, under MRI guidance. Preferential stimulation of left and right hemisphere was achieved by orienting the coil laterally, with the current flow directed towards the hemisphere to be stimulated. Resting motor thresholds were established based on activation of the contralateral extensor halluces longus (EHL), determined by visual inspection.

During treatment, rTMS was delivered over the dmPFC scalp coordinate above, with lateral coil orientation for preferential stimulation of the left then right dmPFC at 120% of the EHL resting motor threshold, at a stimulation frequency of 10 Hz, with a duty cycle of 5 s on and 10s off, for 60 trains, resulting in 3000 pulses per hemisphere in each session. Stimulation was delivered on weekdays for 20 daily sessions on weekdays, with non-remitters offered extension to 30 sessions.

*Neuroimaging Acquisition and Analysis*

Patients underwent MRI sessions one week prior to and one week after rTMS treatment, on a 3T GE Signa HDx scanner equipped with an 8-channel phased-array head coil, using a protocol we have previously reported(Salomon*s et* al, 2014). This comprised a T1-weighted fast spoiled gradient-echo anatomical scan (TE=12ms, TI=300ms, flip angle=20**°**, 116 sagittal slices, thickness=1.5mm, no gap, 256x256 matrix, FOV=240mm), followed by a 10-min resting-state, eyes-closed T2* series (TE=30ms, TR2000ms, flip angle=85**°**, 32 axial slices, thickness=5mm, no gap, 64x64 matrix, FOV=220mm, 300 TRs, 2 s temporal resolution).

MRI data preprocessing and seed-based region-of-interest analyses also followed previously reported methods(Salomon*s et* al, 2014), implemented in the FSL software package(Jenkinso*n et* al, 2012), using FEAT(Beckman*n et* al, 2003) for the following steps: discarding the first 5 volumes for signal stabilization, interleaved slice-timing correction, tissue segmentation using BET(Smith, 2002), motion correction using MCFLIRT(Jenkinso*n et* al, 2002), and spatial smoothing (6 mm FWHM Gaussian kernel). A nuisance linear regression analysis was conducted using 6 motion parameters and FAST(Zhan*g et* al, 2001) extracted white matter and cerebrospinal fluid mean time series. Functional data was then bandpass filtered (0.009-0.009 Hz) and co-registered to the MNI-152 standard atlas.

One cortical and five subcortical regions of interest (ROI) were selected as seeds for whole-brain functional connectivity analysis. In cortex, a dmPFC region of interest (ROI) were defined from a resting-state connectivity-based atlas(Craddoc*k et* al, 2012), based on proximity to the rTMS stimulation coordinate. Two other seeds were selected based on CSTC connectivity in OCD and their therapeutic use in DBS: the medial dorsal thalamus, from an thalamic connectivity atlas(Behren*s et* al, 2003), and the subthalamic nucleus(STN), from a probabilistic STN atlas(Forstman*n et* al, 2012). Three exploratory bilateral ventral striatal sites (superior ventral striatum [VSs], inferior ventral striatum [VSi] and ventral rostral putamen[VRP])(Di Martin*o et* al, 2008) were selected to identify striatal connectivity associated with response. A medial dorsal thalamus (MDT) ROI was created using the Oxford Thalamic Connectivity Atlas(Behren*s et* al, 2003), and a subthalamic nucleus (STN) ROI was created using a probabilistic subthalamic nucleus atlas(Forstman*n et* al, 2012).

Each seed ROI was co-registered from standard space to each subject’s anatomical MRI using the transformation matrix from the original registration to standard space in FLIRT, and its mean time series was then extracted and used as a regressor in a first-level analysis to generate a whole-brain map of voxels positively and negatively correlated to each ROI for each subject. To localize regions where pre-treatment functional connectivity correlated to treatment response, FSL’s FLAME mixed effects model(Beckman*n et* al, 2003) was then applied for the group-level analysis using the responder/non-responder status of each subject as a categorical, group-level regressor.

To localize regions where the *changes* in functional connectivity from pre- to post-treatment correlated to the degree of treatment response, a within-subjects, fixed-effects general linear model analysis was performed for each subject and seed region. This resulted in a set of first-level statistical parametric maps for each subject and each seed indicating regional increases and decreases in functional connectivity to the seed from pre- to post-treatment. For group-level analysis, these individual-subject change maps were then entered into a between-subjects mixed-effects linear regression analysis, using the responder/non-responder status of each subject as a categorical, group-level independent variable. Corrections for multiple comparisons were performed using Gaussian random field theory (Z>1.96, cluster significance p<0.05 corrected).

Parameter estimates for individual subjects’ functional connectivity values (mean z-score) of our seed and cluster regions were then extracted for *post hoc* analysis. An 8 mm sphere centered at the peak cluster voxel (masked by the relevant cluster to ensure anatomical specificity) was registered from standard space to each individual subject using the transformation matrix from the original registration, extracting the mean z-score values from relevant connectivity maps. Baseline z-scores were extracted from individual subjects’ baseline functional connectivity maps. Change z-scores were extracted from individual subjects’ pre-versus-post-treatment contrast maps.

**Supplemental Results**

*fMRI Predictors of Treatment Response*

From the ventral striatal (VRP) seed, responders were characterized by significantly higher pre-treatment functional connectivity to the left dorsolateral and dorsomedial prefrontal cortex (Figure S1A), and significantly lower functional connectivity to the right posterior insula, superior temporal gyrus, supramarginal gyrus, pre-central gyrus and post-central gyrus (Table S2). Baseline VRP-dmPFC connectivity was significantly higher in responders than in non-responders (t_18_=2.2, p=0.04), or healthy controls (t_48_=2.02, p=0.05)(Figure S1B). Of note, higher baseline VRP-dmPFC connectivity significantly predicted a greater percent improvement in YBOCS score following treatment (Pearson *r*_17_=0.51, p=0.03). Baseline VRP-dmPFC connectivity was positive in responders (z=2.81±0.80), slightly negative in non-responders (z=-0.62±1.33) and positive in healthy controls(z=1.20±0.34).

From the thalamic (MDT) seed, responders were characterized by significantly higher pre-treatment functional connectivity to the bilateral pregenual cingulate, posterior cingulate and precuneus, and medial/lateral orbitofrontal cortex, and lower connectivity to the bilateral insula, temporal pole, left hippocampus and pre-central gyrus (Table S2, Figure S1C). Baseline MDT-right OFC functional connectivity was significantly higher in responders versus non-responders (t_18_=2.53, p=0.02)(Figure S1D), and significantly predicted percent improvement in YBOCS score following treatment (*r*_17_=0.56, p=0.01). Likewise, baseline MDT-left OFC connectivity was also significantly higher in responders versus non-responders (t_18_=2.58, p=0.02) and significantly predicted percent YBOCS improvement (*r*_17_=0.63, p=0.004). MDT-left OFC connectivity was positive in responders (z=0.70±0.75) and negative in non-responders (z=-2.13±0.36). Neither group differed significantly from healthy controls in MDT-left OFC or right OFC connectivity.

For the subthalamic nucleus (STN) seed, responders were characterized by significantly higher pre-treatment functional connectivity to the bilateral thalamus and caudate nucleus, and lower functional connectivity to the bilateral insula, supplementary motor area, right pre-central gyrus and left temporal pole (Table S2, Figure S1E). Baseline STN-thalamus functional connectivity was significantly higher in responders versus non-responders (t_18_=2.36, p=0.03)(Figure S1F) and significantly predicted percent YBOCS improvement (*r*_17_=0.57, p=0.01). STN connectivity to the thalamus was positive in both responders (z=6.35±0.73) and non-responders (z=2.61±0.84). Likewise, baseline STN-caudate connectivity also significantly predicted percent YBOCS improvement (*r*_17_=0.48, p=0.04). STN connectivity to the caudate nucleus was positive in responders (z=1.73±1.08) and negative in non-responders (z=-1.65±0.56). Neither group differed significantly from healthy controls in STN-caudate and STN-thalamus connectivity.

For the inferior ventral striatum (VSi) seed, responders had significantly higher pre-treatment functional connectivity to the bilateral precuneus and posterior cingulate gyrus, and lower functional connectivity to the superior posterior cingulate gyrus, right supramarginal gyrus and temporal pole relative to non-responders (Table S2). For the superior ventral striatum (VSs) seed, responders had significantly higher functional connectivity to the precuneus and posterior cingulate gyrus, and lower connectivity to the superior temporal gyrus and superior posterior cingulate gyrus (Table S2).

*fMRI Correlates of Treatment Response*

For the VRP seed, increases in functional connectivity to the bilateral brainstem, thalamus and insula and decreases in functional connectivity to the bilateral dmPFC were associated with successful response to treatment (Table S3). Compared to healthy controls, responders’ VRP-dmPFC connectivity was significantly higher at baseline (control z=0.57±0.31, t_48_=2.12, p=0.04) but significantly lower after treatment (t_47_=2.61, p=0.01), with significant reductions in VRP-dmPFC functional connectivity over treatment (pre-treatment z=2.47±0.76, post-treatment z=-2.05±0.81, t_9_=3.99, p=0.004). In contrast, non-responders did not show significantly higher VRP-dmPFC connectivity than healthy controls at baseline (t_45_=0.50, p = 0.68), and did not show any significant change in VRP-dmPFC connectivity over the course of treatment (pre-treatment z=0.45±1.09, post-treatment z=1.35±0.71, t_6_=0.88, p=0.41). However, they did continue to show significantly higher VRP-dmPFC connectivity than both healthy controls and responders after the course of treatment (t_45_=2.45, p=0.02). Across all subjects, the degree of reduction in VRP-dmPFC functional connectivity significantly correlated with percent YBOCS improvement (*r*_15_=-0.64, p=0.008)

For the VSs seed, increased functional connectivity to the bilateral insula and temporal pole and decreased functional connectivity to the bilateral dmPFC were associated with successful treatment response (Table S3). In responders, VSs-dmPFC connectivity decreased significantly from pre- to post-treatment (pre-treatment z=1.33±1.29, post-treatment z=-1.92±0.80, t_8_=2.75, p=0.03), and was significantly lower than in healthy controls following treatment (control z=1.03±0.39, t_47_=2.87, p=0.01).

Conversely, in non-responders, VSs-dmPFC functional connectivity showed a non-significant trend toward increasing from pre- to post-treatment (pre-treatment z=0.21±1.05, post-treatment z=1.79±0.60, t_6_=1.40, p=0.21), and did not differ significantly from healthy controls following treatment (t_45_=0.54, p=0.61). Once again, across all subjects, the degree of reduction in VSs-dmPFC functional connectivity significantly correlated to percent YBOCS improvement (*r*_15_=-0.65, p=0.007).

For the VSi, treatment response was associated with increased connectivity to the right middle temporal gyrus and posterior insula and decreased connectivity to the left occipital pole and cuneus (Table S3). For the MDT, treatment response was associated with increased functional connectivity to the left putamen, amygdala, hippocampus, and parahippocampal gyrus, and decreased functional connectivity to the left caudate and bilateral cuneus and precuneus (Table S3). For the STN, treatment response was associated with decreased functional connectivity to the right precuneus and posterior cingulate cortex (Table S3).

**Figure S1.** High baseline frontal-striatal-thalamic-subthalamic connectivity predicts response to dmPFC-rTMS in OCD. Bar graphs are intended to convey the absolute magnitudes of the parameter estimates in each group, as complementary information for the difference maps. **A.** Regions of higher baseline functional connectivity (orange) to the bilateral VRP seed (green) in rTMS-responders vs. non-responders. **B.** Parameter estimates between the VRP and dmPFC for baseline healthy control, responder and non-responder groups. **C.** Regions of higher baseline functional connectivity (orange) to the bilateral MDT seed (green) in rTMS-responders vs. non-responders. **D.** Parameter estimates between the MDT and OFC for baseline healthy control, responder and non-responder groups. **E.** Regions of higher baseline functional connectivity (orange) to the bilateral STN in rTMS responders vs. non-responders. **F.** Parameter estimates between the STN and Thalamus for baseline healthy control, responder and non-responder groups.

**Table S1:** Centre of gravity coordinates for regions of interest created from parcellation atlases, and MNI coordinates for sphere-based regions of interest.

| Seed | Type of Seed | Citation | MNI | | |
| --- | --- | --- | --- | --- | --- |
|  |  |  | X | Y | Z |
| dmPFC | Parcellation | Craddock, 2012 | 0 | 38 | 24 |
| STN | Parcellation | Forstmann, 2012 | 0 | -12 | -8 |
| MDT | Parcellation | Behrens, 2003 | 2 | -22 | 10 |
| VSs | Coordinate | di Martino, 2008 | 10 | 15 | 0 |
| Vsi | Coordinate | di Martino, 2008 | 9 | 9 | -8 |
| VRP | Coordinate | di Martino, 2008 | 20 | 12 | -3 |

dmPFC = dorsomedial prefrontal cortex; MDT = medial dorsal thalamus; MNI = Montreal Neurological Institute; STN = subthalamic nucleus; VRP = ventral rostral putamen; VSi = inferior ventral striatum; VSs = superior ventral striatum

**Table S2**: Brain regions where baseline resting-state functional connectivity to exploratory seed regions differed significantly between responders and non-responders. All activations are whole-brain Gaussian random field theory corrected for multiple comparisons at a cluster threshold p<0.05, but do not meet across-seed FDR-correction.

| **Seed** | **Region** | | **Brodmann Area** |  | **MNI Coordinate** | | | **Peak Z Score** |
| --- | --- | --- | --- | --- | --- | --- | --- | --- |
|  |  |  |  |  | X | Y | Z |  |
| **MDT** |  | ***Response > Nonresponse*** |  |  |  |  |  |  |
|  | B | Pregenual Cingulate | 9 |  | -2 | 46 | 12 | 3.05 |
|  | L | Medial, Lateral OFC | 10, 11, 25 |  | -12 | 26 | -22 | 3.35 |
|  | R | Lateral OFC | 10, 11 |  | 40 | 50 | -6 | 3.38 |
|  | B | Posterior Cingulate Gyrus | 23, 30, 31 |  | -2 | -66 | 12 | 3.70 |
|  | B | Precuneus | 7, 31 |  | 8 | -70 | 38 | 4.18 |
|  |  |  |  |  |  |  |  |  |
|  |  | ***Nonresponse > Response*** |  |  |  |  |  |  |
|  | R | Temporal Pole, Superior/Middle Temporal Gyrus | |  | 52 | -14 | -8 | 3.50 |
|  | L | Temporal Pole | 22 |  | -56 | 8 | -2 | 3.65 |
|  | L | Precentral Gyrus | 3, 4 |  | -54 | -8 | 40 | 3.77 |
|  | L | Insula | 13 |  | -36 | -8 | -8 | 3.91 |
|  | R | Insula | 13 |  | 36 | 6 | -4 | 2.51 |
|  | L | Hippocampus |  |  | -34 | -12 | -20 | 2.90 |
| **STN** |  | ***Response > Nonresponse*** |  |  |  |  |  |  |
|  | R | Thalamus |  |  | 16 | -22 | 4 | 3.99 |
|  | L | Thalamus |  |  | -4 | -22 | 14 | 4.06 |
|  | R | Caudate Nucleus |  |  | 14 | 10 | 14 | 3.36 |
|  | L | Caudate Nucleus |  |  | -18 | 16 | 10 | 3.13 |
|  |  |  |  |  |  |  |  |  |
|  |  | ***Nonresponse > Response*** |  |  |  |  |  |  |
|  | L | Temporal Pole, Inferior Frontal Gyrus |  |  | -42 | 18 | 16 | 4.02 |
|  | L | Insula | 13 |  | -48 | 2 | -8 | 3.94 |
|  | B | SMA |  |  | -10 | 8 | 64 | 3.50 |
|  | R | Precentral Gyrus |  |  | 34 | -12 | 58 | 4.53 |
|  | R | Insula | 13 |  | 44 | -12 | 8 | 3.06 |
| **VRP** |  | ***Response > Nonresponse*** |  |  |  |  |  |  |
|  | L | Dorsomedial Prefrontal Cortex | 9, 32 |  | -10 | 32 | 26 | 3.19 |
|  | L | Dorsolateral Prefrontal Cortex | 6 |  | -30 | 16 | 42 | 3.26 |
|  |  |  |  |  |  |  |  |  |
|  |  | ***Nonresponse > Response*** |  |  |  |  |  |  |
|  | R | Posterior Insula | 13 |  | 38 | -8 | 8 | 3.77 |
|  | R | Posterior Superior Temporal Gyrus | 41 |  | 58 | -20 | 8 | 3.62 |
|  | R | Supramarginal Gyrus | 40 |  | 62 | -20 | 22 | 3.20 |
|  | R | Pre/Postcentral Gyrus | 3, 4 |  | 60 | 12 | 26 | 3.91 |
| **VSi** |  | ***Response > Nonresponse*** |  |  |  |  |  |  |
|  | B | Precuneus | 23, 31 |  | -4 | -58 | 28 | 3.05 |
|  | B | Posterior Cingulate Gyrus | 30, 31 |  | -4 | -42 | 18 | 3.10 |
|  |  |  |  |  |  |  |  |  |
|  |  | ***Nonresponse > Response*** |  |  |  |  |  |  |
|  | R | Supramarginal Gyrus | 40 |  | 64 | -20 | 28 | 5.60 |
|  | B | Dorsal Posterior Cingulate Gyrus | 31 |  | 6 | -20 | 40 | 4.44 |
|  | R | Temporal Pole | 22 |  | 62 | 10 | -2 | 4.40 |
|  |  |  |  |  |  |  |  |  |
| **VSs** |  | ***Response > Nonresponse*** |  |  |  |  |  |  |
|  | B | Precuneus, PCC | 23, 30, 31 |  | -6 | -56 | 16 | 3.24 |
|  |  |  |  |  |  |  |  |  |
|  |  | ***Nonresponse > Response*** |  |  |  |  |  |  |
|  | R | STS | 40, 42 |  | 58 | -30 | 10 | 3.97 |
|  | L | STS | 40, 42 |  | -56 | -32 | 14 | 3.20 |
|  | R | Posterior Cingulate Gyrus | 31 |  | 6 | -16 | 50 | 3.79 |

MDT=medial dorsal thalamus; MNI=Montreal Neurological Institute; OFC=orbitofrontal cortex; SMA=supplementary motor area; STN=subthalamic nucleus; VRP=ventral rostral putamen; VSi=inferior ventral striatum; VSs=superior ventral striatum

**Table S3**: Brain regions where the pre-to-post treatment change in functional connectivity to exploratory that differed significantly between rTMS responders and non-responders. All activations are Gaussian random field theory corrected for multiple comparisons at a cluster threshold p<0.05. All activations are whole-brain Gaussian random field theory corrected for multiple comparisons at a cluster threshold p<0.05, but do not meet across-seed FDR-correction.

| **Seed** |  | **Region** | **Brodmann Area** |  | **MNI Coordinate** | | | **Peak Z Score** |
| --- | --- | --- | --- | --- | --- | --- | --- | --- |
|  |  |  |  |  | **X** | **Y** | **Z** |  |
| **MDT** |  | ***FC increase in Resp > Nonresp*** | |  |  |  |  |  |
|  | L | Putamen |  |  | -32 | -16 | 10 | 2.82 |
|  | L | Amygdala, Hippocampus | |  | -26 | -4 | -16 | 2.8 |
|  | L | Parahippocampal Gyrus | |  | -18 | -24 | -22 | 3.36 |
|  |  |  |  |  |  |  |  |  |
|  |  | ***FC reduction in Resp > Nonresp*** | |  |  |  |  |  |
|  | L | Caudate Nucleus | |  | -20 | 10 | 16 | 3.57 |
|  | B | Cuneus, Precuneus | |  | 0 | -72 | 26 | 3.33 |
|  | L | Intracalcarine Cortex | 30 |  | -10 | -70 | 16 | 3.29 |
| **STN** |  | ***FC reduction in Resp > Nonresp*** | |  |  |  |  |  |
|  | R | Precuneus, PCC | 31 |  | 12 | -62 | 32 | 3.85 |
| **VRP** |  | ***FC increase in Resp > Nonresp*** | |  |  |  |  |  |
|  | B | Brainstem |  |  | -2 | -22 | -24 | 3.1 |
|  | L | Thalamus |  |  | -8 | -26 | 0 | 2.72 |
|  | R | Thalamus |  |  | 12 | -26 | 0 | 3.08 |
|  | L | Insula |  |  | -36 | -4 | 2 | 3.04 |
|  | R | Insula |  |  | 42 | 2 | -10 | 3.57 |
|  |  |  |  |  |  |  |  |  |
|  |  | ***FC reduction in Resp > Nonresp*** | |  |  |  |  |  |
|  | B | Dorsomedial Prefrontal Cortex | 32 |  | -10 | 18 | 42 | 3.62 |
|  | L | Supracalcarine Cortex | 17 |  | -4 | -90 | 10 | 3.86 |
|  | R | Occipital Pole | 19 |  | -4 | -98 | -2 | 3.75 |
| **VSi** |  | ***FC increase in Resp > Nonresp*** | |  |  |  |  |  |
|  | R | Middle Temporal Gyrus (Posterior) | 12 |  | 62 | -36 | -10 | 3.76 |
|  | R | Posterior Insula | 13 |  | 40 | -4 | -10 | 3.06 |
|  |  |  |  |  |  |  |  |  |
|  |  | ***FC reduction in Resp > Nonresp*** | |  |  |  |  |  |
|  | L | Occipital Pole | 18 |  | -6 | -96 | -6 | 4.4 |
|  | L | Cuneus | 7, 19 |  | -8 | -88 | 38 | 3.67 |
| **VSs** |  | ***FC increase in Resp > Nonresp*** | |  |  |  |  |  |
|  | L | Insula | 13 |  | -38 | 0 | -8 | 2.95 |
|  | L | Temporal Pole, Amygdala | 34 |  | -38 | 4 | -20 | 3.64 |
|  | R | Temporal Pole, Inferior Temporal Gyrus | 20 |  | 48 | -32 | -20 | 3.22 |
|  | R | Insula | 13 |  | 40 | 2 | -10 | 3.64 |
|  |  |  |  |  |  |  |  |  |
|  |  | ***FC reduction in Resp > Nonresp*** | |  |  |  |  |  |
|  | B | Dorsomedial Prefrontal Cortex | 32 |  | -8 | 18 | 42 | 4.51 |
|  | B | Dorsomedial Prefrontal Cortex | 9, 32 |  | -10 | 30 | 30 | 4.35 |

FC=functional connectivity; MDT=medial dorsal thalamus; MNI=Montreal Neurological Institute; OFC=orbitofrontal cortex; PCC= posterior cingulate cortex; SMA=supplementary motor area; STN=subthalamic nucleus; VRP=ventral rostral putamen; VSi=inferior ventral striatum; VSs=superior ventral striatum

**References**

Beckmann CF, Jenkinson M, Smith SM (2003). General multilevel linear modeling for group analysis in FMRI. *Neuroimage* **20**: 1052–1063.

Behrens TEJ, Johansen-Berg H, Woolrich MW, Smith SM, Wheeler-Kingshott CAM, Boulby PA, *et al* (2003). Non-invasive mapping of connections between human thalamus and cortex using diffusion imaging. *Nat Neurosci* **6**: 750–757.

Craddock RC, James GA, Holtzheimer PE, Hu XP, Mayberg HS (2012). A whole brain fMRI atlas generated via spatially constrained spectral clustering. *Hum Brain Mapp* **33**: 1914–28.

Forstmann BU, Keuken MC, Jahfari S, Bazin PL, Neumann J, Schäfer A, *et al* (2012). Cortico-subthalamic white matter tract strength predicts interindividual efficacy in stopping a motor response. *Neuroimage* **60**: 370–375.

Jenkinson M, Bannister P, Brady M, Smith S (2002). Improved optimization for the robust and accurate linear registration and motion correction of brain images. *Neuroimage* **17**: 825–841.

Jenkinson M, Beckmann CF, Behrens TEJ, Woolrich MW, Smith SM (2012). FSL. *Neuroimage* **62**: 782–790.

Martino a Di, Scheres a, Margulies DS, Kelly a MC, Uddin LQ, Shehzad Z, *et al* (2008). Functional connectivity of human striatum: a resting state FMRI study. *Cereb Cortex* **18**: 2735–47.

Salomons T V, Dunlop K, Kennedy SH, Flint A, Geraci J, Giacobbe P, *et al* (2014). Resting-State Cortico-Thalamic-Striatal Connectivity Predicts Response to Dorsomedial Prefrontal rTMS in Major Depressive Disorder. *Neuropsychopharmacology* **39**: 488–98.

Smith SM (2002). Fast robust automated brain extraction. *Hum Brain Mapp* **17**: 143–155.

Zhang Y, Brady M, Smith S (2001). Segmentation of brain MR images through a hidden Markov random field model and the expectation-maximization algorithm. *IEEE Trans Med Imaging* **20**: 45–57.
