# Supplementary material for: Reductions in Cortico-Striatal Hyperconnectivity Accompany Successful Treatment of Obsessive-Compulsive Disorder with Dorsomedial Prefrontal rTMS
Source: Neuropsychopharmacology. 2015 Oct 28;41(5):1395–403. doi: 10.1038/npp.2015.292 (PMC4793124; doi:10.1038/npp.2015.292)
Supplement: Supplementary Figures [file npp2015292x2.ppt]

## Slide 1
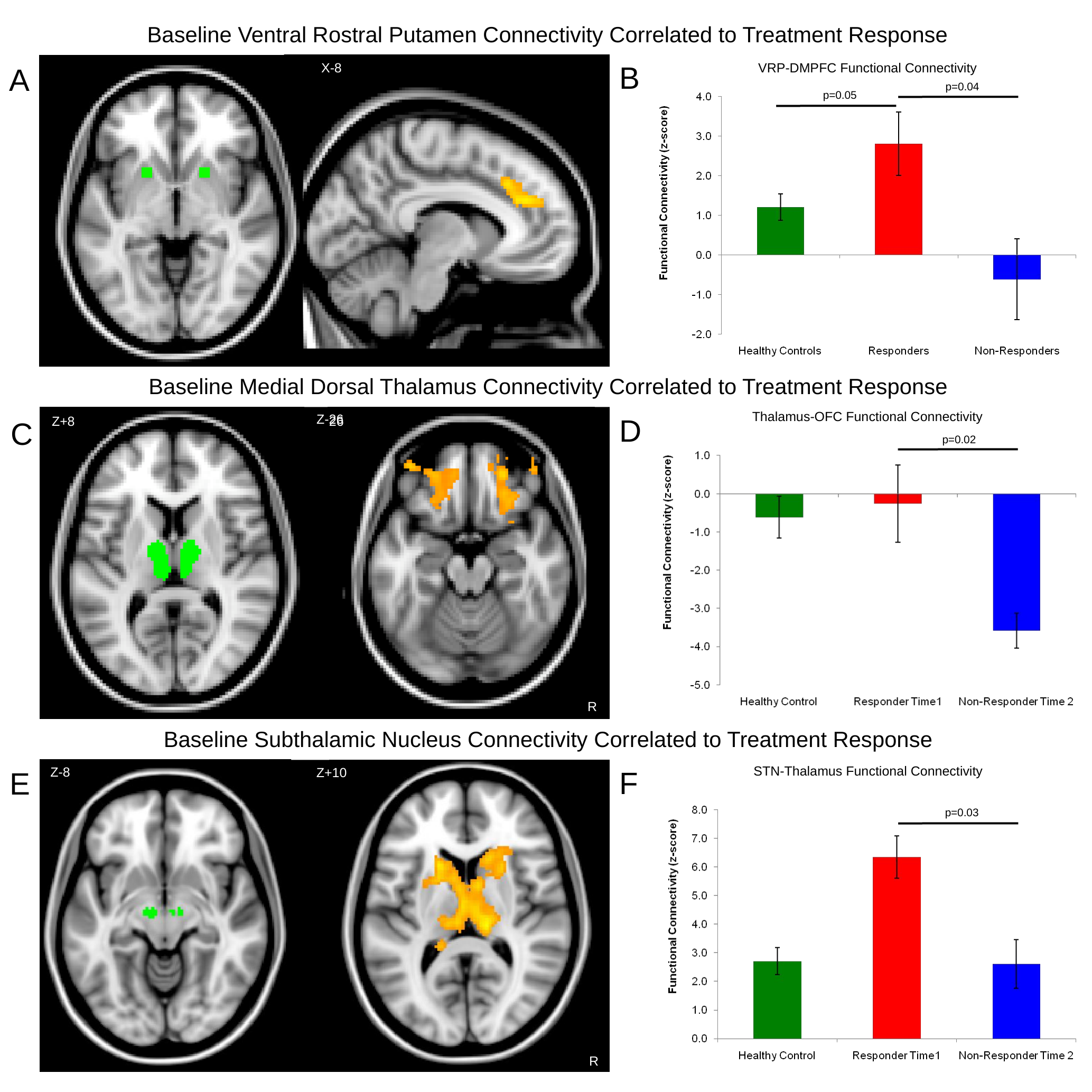

Baseline Ventral Rostral Putamen Connectivity Correlated to Treatment Response
B
B
Z-4
X-8
VRP-DMPFC Functional Connectivity
A
p=0.04
p=0.05
R
Baseline Medial Dorsal Thalamus Connectivity Correlated to Treatment Response
Thalamus-OFC Functional Connectivity
Z-26
D
Z+8
Z-26
C
p=0.02
R
R
Baseline Subthalamic Nucleus Connectivity Correlated to Treatment Response
STN-Thalamus Functional Connectivity
F
Z-8
E
Z+10
p=0.03
R
